# Studying Lactoferrin *N*-Glycosylation

**DOI:** 10.3390/ijms18040870

**Published:** 2017-04-20

**Authors:** Sercan Karav, J. Bruce German, Camille Rouquié, Annabelle Le Parc, Daniela Barile

**Affiliations:** 1 Department of Molecular Biology and Genetics, Canakkale Onsekiz Mart University, 17100 Canakkale, Turkey; sercankarav@gmail.com; 2 Department of Food Science and Technology, University of California, One Shields Avenue, Davis, CA 95616, USA; camille.rouquie@hotmail.fr (C.R.); alebouedec@prolacta.com (A.L.P.); dbarile@ucdavis.edu (D.B.); 3 Foods for Health Institute, University of California, One Shields Avenue, Davis, CA 95616, USA

**Keywords:** lactoferrin, *N*-glycans, deglycosylating enzymes, mass spectrophotometry, bioinfomatic libraries, structure-activity studies

## Abstract

Lactoferrin is a multifunctional glycoprotein found in the milk of most mammals. In addition to its well-known role of binding iron, lactoferrin carries many important biological functions, including the promotion of cell proliferation and differentiation, and as an anti-bacterial, anti-viral, and anti-parasitic protein. These functions differ among lactoferrin homologs in mammals. Although considerable attention has been given to the many functions of lactoferrin, its primary nutritional contribution is presumed to be related to its iron-binding characteristics, whereas the role of glycosylation has been neglected. Given the critical role of glycan binding in many biological processes, the glycan moieties in lactoferrin are likely to contribute significantly to the biological roles of lactoferrin. Despite the high amino acid sequence homology in different lactoferrins (up to 99%), each exhibits a unique glycosylation pattern that may be responsible for heterogeneity of the biological properties of lactoferrins. An important task for the production of biotherapeutics and medical foods containing bioactive glycoproteins is the assessment of the contributions of individual glycans to the observed bioactivities. This review examines how the study of lactoferrin glycosylation patterns can increase our understanding of lactoferrin functionality.

## 1. Introduction

Lactoferrin is a highly glycosylated protein that was first isolated from bovine milk in 1939 by Sorensen and Sorensen [1], and later identified in human milk in 1960 by Johanson [2]. It has been identified in secretions from exocrine glands as well as in specific granules of neutrophils [3]. Lactoferrin is present in large amounts in milk, and in mammalian exocrine secretions such as saliva, tears, mucus, white blood cells, seminal fluid, and bronchial secretions [4,5]. Lactoferrin content in milk varies depending on the mammalian species and the stage of lactation [6]. Lactoferrin is the second most abundant whey protein in human milk, with a concentration of 2–4 mg/mL (6–8 mg/mL in colostrum) [7]. Its concentration in bovine milk ranges between 0.02 and 0.2 mg/mL, and between 0.2 and 2 mg/mL in pig, mouse, and horse milk, whereas rat, rabbit, and dog milks contain less than 0.05 mg lactoferrin/mL [8]. Oral administration of lactoferrin has been proposed to exert various beneficial health effects in humans and animals, including anti-cancer, anti-inflammatory, and anti-infective activities [9,10,11]. Lactoferrin is also used for the prevention of lipid oxidation and the improvement of microflora [12,13].

Milk lactoferrin possesses varying numbers of potential glycosylation sites, depending on the species of origin [14]. Although the contribution of conjugated glycans to the functions of lactoferrin is not fully understood, some connections between glycans and physicochemical and biological roles of lactoferrin have been reported [15,16,17,18]. Lactoferrin has been produced from various microorganisms, transgenic animals (cows, goats) and recombinant plants, and through large-scale isolation methods from bovine cheese whey [19,20]. Cation-exchange chromatography is the most common method used to isolate lactoferrin from dairy products. Lactoferrin exhibits anti-oxidant and anti-microbial properties, and has many applications in the food industry. For example, bovine lactoferrin (bLF) is used to supplement food products such as cakes, pastries, yogurts, and drinks, and non-food products such as cosmetics. To develop lactoferrin-enhanced foods, and infant formulas in particular, a natural source of lactoferrin from dairy milk is used. An appropriate design of infant formula requires the addition of lactoferrin that mimics the properties of human milk lactoferrin, and improves the immune responses of newborns. bLF is also approved by the Food and Drug Administration in the USA as an ingredient in anti-microbial sprays for use on uncooked beef carcasses to eliminate pathogens and extend shelf life [21]. Lactoferrin is used to inhibit lipid oxidation due its iron-binding capacity, as iron-promoted lipid oxidation is responsible for rancidity, and decreases the shelf life of commercial products, including infant formula and skincare cosmetics [22].

## 2. Characteristics of Lactoferrin

Lactoferrin—also known as lactotransferrin—is comprised of 692 amino acids folded into two globular lobes that are connected by an α–helix. It is a member of the transferrin family (60% amino acid sequence identity with serum transferrin) with a high capability of binding and transferring Fe^3+^ ions [23]. Lactoferrin and transferrin have similar amino acid compositions, secondary structures, (including their disulfide linkages), and tertiary structures, although they differ in terms of biological functions [24,25]. Glycans attached to lactoferrin are complex and more heterogeneous than those attached to other transferrins. The heterogeneity and complexity of the glycans are believed to be the basis for at least a part of the differences in the respective biological properties of lactoferrins [23].

Three different isoforms of lactoferrin have been identified: lactoferrin-α, lactoferrin-γ, and lactoferrin-β. Lactoferrin-α is the iron-binding form. Lactoferrin-γ and lactoferrin-β have ribonuclease activity, and do not bind iron [26]. Iron-binding sites exist on each lobe in lactoferrin-α with two domains; C_1_, C_2_, N_1_ and N_2_ domains. Histidine, tyrosine, and aspartic acid are the most important amino acids for iron binding [27]. Lactoferrin is the only transferrin capable of retaining Fe^3+^ ions in a wide range of pH values [7]. In addition to high iron-binding capacity, lactoferrin is also capable of binding to a wide variety of compounds, including DNA, lipopolysaccharides, heparin, and metal ions including Al^3+^, Mn^3+^, Cu^2+^, and Zn^2+^ [27].

Lactoferrin displays high resistance to proteolytic degradation by trypsin-like enzymes, though its digestion is proportional to the degree of iron saturation. This resistance to proteolysis results in only partial digestion in the gut [28,29], and iron-saturated lactoferrin (i.e., halolactoferrin) is more resistant than the iron-depleted form (apolactoferrin) [29]. Abnormal *N*-glycan composition decreases the resistance of lactoferrins to proteolysis, and alters their immunogenicity [30].

The effects of various heat treatments on the stability of lactoferrin and its biological functions have been widely studied. Abe et al. [31] concluded that 15 seconds of pasteurization at 72 °C did not significantly affect lactoferrin, whereas ultra-high temperature (UHT) pasteurization at 135 °C for four seconds resulted in the loss of iron-binding ability and anti-microbial characteristics. Also, apolactoferrin was denatured faster than halolactoferrin. Oria et al. [32] showed that heat treatment of lactoferrin under industrial processing conditions did not significantly affect its interaction with monocytic cells, an interaction that is the rationale for its use in infant formulas. Brisson et al. [33] demonstrated that the binding of iron by lactoferrin improved its thermal stability and its isolation from whey. Upon high heat treatment, lactoferrin aggregates with cysteine-containing proteins (к-casein, α-lactalbumin, and β-lactoglobulin) by thiol/disulfide-exchange reactions that lower its recovery from whey.

## 3. Biological Roles of Lactoferrin

**Figure 1:**
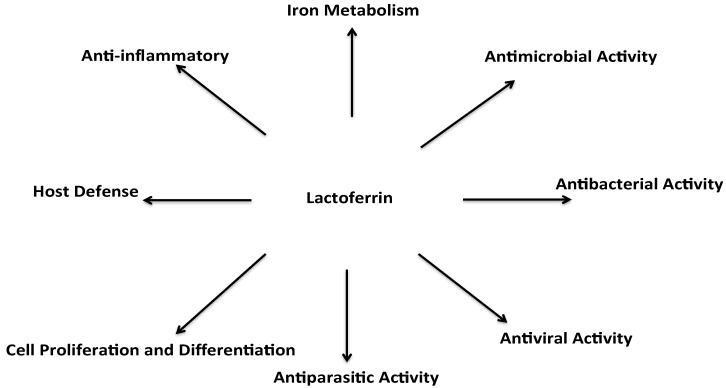
Biological roles of lactoferrin (adapted from Brock) [42].

Lactoferrin has attracted much scientific and industrial interest over the past fifty years due to its various biological functions, including bifidogenic, anti-microbial [34], immunomodulatory [35], anti-inflammatory [36], and anti-carcinogenic activities [37,38] (Figure 1). The anti-microbial activity of lactoferrin is based on two mechanisms; the depletion of iron (an essential nutrient for all microorganisms), and direct interaction with pathogens that cause cell lysis [39]. Lactoferrin reduces the growth of a wide variety of microorganisms, including bacteria, fungi, viruses, and protozoa. Especially, its ability to bind iron, at even at low pHs fueled by bacterial metabolism, prevents bacterial proliferation. Qui et al. showed that human lactoferrin inactivates colonization factors from *Haemophilus influenza* [34]. Moreover, Jahani et al. demonstrated that lactoferrin is effective against both Gram-positive (*Staphylococcus epidermis*, *Bacillus cereus*) and Gram-negative (*Campylobacter jejuni*, *Salmonella*) bacteria. However, it was shown that lactoferrin is more active as antimicrobial agent on Gram-positive bacteria than on Gram-negative bacteria.

Interestingly, lactoferrin acts as a selective anti-microbial by killing pathogens, and stimulating the growth of beneficial microorganism such as *Lactobacillus* and *Bifidobacteium.* Petschow et al. [40] demonstrated that blF specifically promotes the growth of *B. infantis* and *B. breve*, whereas human lactoferrin (hLF) stimulates greater growth of *B. infantis* in vitro. Karav et al. have also shown that glycans consisting of the five monosaccharides—hexose (Hex), *N*-acetylglucosamines (GlcNAc), fucose (Fuc), sialic acid (NeuAC), and *N*-glycolylneuraminic acid (NeuGc)—released from cheese whey, or lactoferrin-rich colostrum, selectively stimulated the growth of *B. infantis* [41]. Especially, the glycan compositions 4Hex-3HexNAc-1Fuc, 3Hex-5HexNAc, 5Hex-2HexNAc-1NeuAc, 5Hex-4HexNAc-1NeuAc, and 5Hex-3HexNAc-1NeuAc, potentially originating from lactoferrin, were preferentially utilized as substrates by *B. infantis*. Lactoferrin also plays important roles in immune host defense by supporting the proliferation, differentiation, and activation of immune system cells [14].

## 4. Lactoferrin Sources

In an attempt to increase lactoferrin concentration in ruminant milks and facilitate lactoferrin production to meet a growing market demand, alternative methods have been explored. The physicochemical and biological roles of recombinant human lactoferrin (rhLF) expressed in fungi, viruses, cell cultures, plants, and animals, including rabbit, goat, bovine, and mouse milks have been widely investigated [43,44,45]. While it is known that gene polymorphisms occur in hLF at amino acid positions 4 (deletion of Arg in a low percentage of people), 11 (Ala or Thr), 29 (Arg or Lys) and 561 (Asp or Glu), the human lactoferrin sequence used to make transgenic cows (containing a genomic hLF sequence) had an Arg at position 4, Ala at 11, Arg at 29 and Asp at 561 [30]. Therefore, the amino acids (number and type) in the primary structure of rhLF produced in bovine milk, were ostensibly the same as that of natural human milk lactoferrin.

rhLF appears to have physicochemical and biological properties similar to those of hLF [44]. Similar to the primary structure of hLF, that of rhLF contains ~700 amino acid residues, and the protein is folded into two lobes. Each lobe can bind an iron atom, and contains potential glycosylation sites. Despite the high structural similarity between rhLF and hLF, rhLF has a slightly lower apparent mass than hLF, which could be due to a variation in their *N*-glycan patterns. The iron-saturated forms of rhLF and hLF have similar crystal structures [19], and rhLF has iron-binding and iron-releasing properties equivalent to those of hLF. The crystallographic structure of rhLF in its iron-saturated conformation was reported as almost identical to the structure of iron-saturated natural hLF [19]. These properties show that rhLF, like hLF [43,44], can potentially protect against microbial and viral attacks. Some studies found that rhLF is more sensitive to proteolysis than hLF [30], possibly as a result of the glycosylation differences between the two proteins.

Although the structure of rhLF expressed in bovine milk closely matches the structure of hLF, there are important differences in glycosylation patterns. Both rhLF produced in cloned cattle and hLF have the same two major sites of glycosylation (Asn138 and Asn479) [19], but the *N*-glycan structures are different. Yu et al. [46] demonstrated that *N*-glycans from rhLF have generally high mannose, hybrid, and complex-type structures, with less NeuAc and fucose, in contrast to hLF, which contains highly sialylated and fucosylated complex structures. Van Berkel et al. [43] showed that hLF contains complex-type glycans, and that rhLF produced in transgenic cows has more oligomannose and hybrid type glycans than does hLF. Because glycosylation is a species-specific and tissue-specific modification system, rhLF may present some glycan patterns that are typical of bovine milk glycome. The bioactivity of rhLf were tested on animals’ models [47], including pigs [48] and neonatal mice [49]. These studies reveal beneficial effects on the microbiome of the animal (decrease of *E. coli* and *Salmonella* and increase of *Bifidobacterium* spp.) and their intestinal growth.

## 5. Glycosylation of Lactoferrin

**Figure 2:**
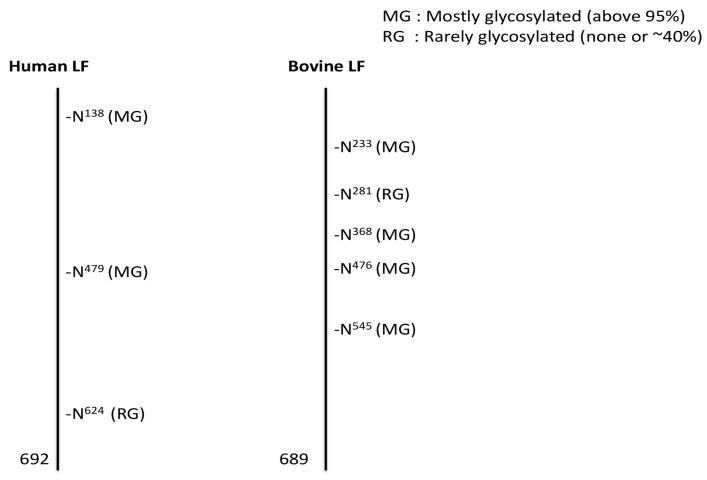
Potential and actual *N*-glycosylation sites of bovine and human lactoferrin (adapted from Van Veen et al. [30]).

Glycosylation is one of the most common and complex forms of protein post-translational modification. More than 50% of eukaryotic proteins are glycosylated, and this glycosylation plays an important role in the protein’s biological function. All lactoferrins identified to date are glycosylated with a varying number of potential glycosylation sites depending on the species [14]. Human lactoferrin contains three potential *N*-glycosylation sites: asparagine (Asn) 138, Asn479, Asn624; caprine, bovine, and ovine lactoferrin have five sites: Asn233, 281, 368, 476, and 545), whereas murine lactoferrin has only one potential *N*-glycosylation site: Asn476 [50]. Among these glycosylation sites, only two sites are commonly glycosylated in hLF: Asn138 and Asn479 [51], and four sites are glycosylated in blF: Asn233, Asn368, Asn476, and Asn545 (Figure 2) [52].

**Figure 3:**
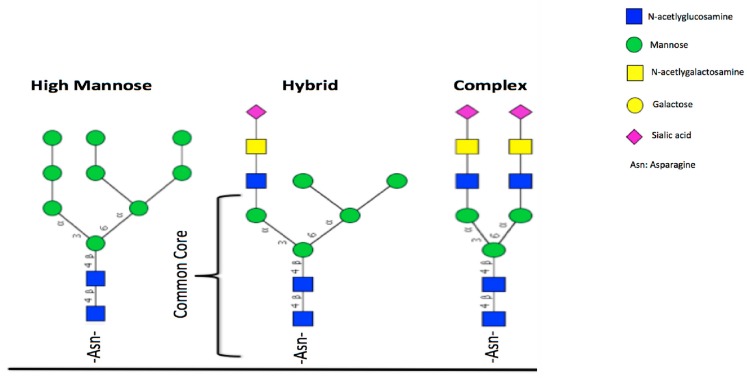
Three types of *N*-glycans (Adapted from *Essentials of Glycobiology*, 2nd edition, 2009) [56].

*N*-glycans are covalently attached to the Asn residue of the lactoferrin protein through an *N*-glycosidic bond if the specific amino acid sequence is Asn-X-Ser/Thr (where X can be any amino acid except proline). The *N*-glycan core is composed of two GlcNAc and three mannoses as shown in Figure 3. The *N*-glycan core is assembled in the endoplasmic reticulum of the cell. The glycan is elongated with other monosaccharides via the action of enzymes, which determines the degree of branching and the type of linkage. Elongation with NeuAC and Fuc increases the diversity and complexity of the *N*-glycan structure [53,54]. *N*-glycans share a common pentasaccharide core region, and can be generally divided into three main classes: oligomannose, complex, and hybrid (Figure 2). High mannose contains only mannose residues attached to the core. Complex *N*-glycans are built with two “antennae” branching from twin GlcNAc molecules attached to the core. Hybrid class *N*-glycans show only mannose residues attached to the Manα1-6 arm, and one or two antennae containing GlcNAc attached to the Manα1-3 arm. GlcNAc branching of complex *N*-glycans leads to more diverse structures than bi-antennary ones. Glycan function varies based on the glycan composition and the site of attachment and this diversity is involved in the functional activity of the protein. Most lactoferrins contain bi-antennary glycans, whereas hLF also possess multiple poly-antennary glycans [55].

**Figure 4:**
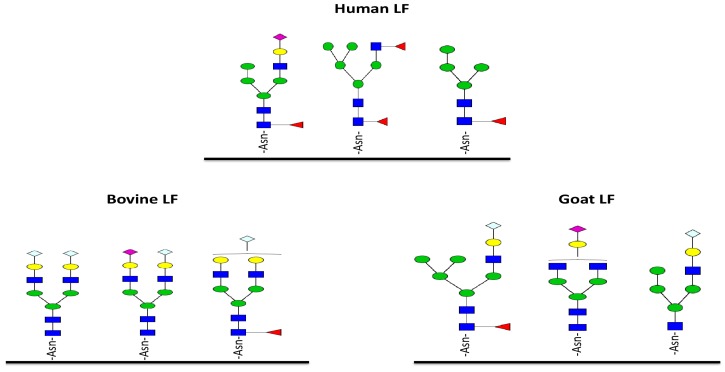
Examples of unique glycans for human, bovine, and goat lactoferrins. Green circles, yellow circles, blue squares, red triangles, purple diamonds, and gray diamonds represent mannose, galactose, *N*-acetlyglucosamine, Fucose, sialic acid, and *N*-glycolylneuraminic acid residues, respectively.

Currently, lactoferrin amino acid sequences have been reported for many species, including human, cow, horse, goat, pig, mouse, sheep, buffalo, camel, zebu, and gorilla [57]. The protein contains nearly 690–711 residues, with a degree of homology ranging from 65 to nearly 100% among species [58]. Despite this high homology, the *N*-glycan profiles of the different mammalian lactoferrins are quite different. Le Parc et al. [59] showed that human, cow, and goat lactoferrin have 13 *N*-glycans in common (such as 5Hex2HexNAc, 6Hex4HexNAc, 4Hex4HexNAc, and 5Hex4HexNAc1NeuAc), whereas human, cow, and goat share 16, 18, and six unique glycan structures, respectively (Figure 4). Additionally, NeuGc is present in the *N*-glycan composition of ruminant species, including goat and bovine lactoferrin, and absent in human lactoferrin [38,59]. Differences were also observed among species, within structures containing fucose and sialic acids. The percentage of fucosylation in human lactoferrin is higher than in goat and bovine lactoferrin. These differences may indicate different biological functions. Indeed, Almond et al. showed that different glycan patterns can impact immunogenicity and allergy. Barboza et al. showed that a there was a decrease in glycosylation in the second week of lactation, followed by an increase in total glycosylation, as well as higher-order fucosylation thereafter [60]. Moreover, Le Parc et al. demonstrated that human lactoferrin produced in transgenic cows displays a different glycoprofile pattern, compared to the natural human lactoferrin. In fact, despite the identical amino acid sequence, the profile of the human lactoferrin recombinantly expressed in cows was closer in glycosidation to bovine milk lactoferrin than to the human counterpart, sharing 16 *N*-glycans with bovine and only nine with human lactoferrin, respectively. These differences are indicative of a strong organism-dependent influence on lactoferrin post-translational modifications, and suggest that the proteins so produced might have different biological roles due to altered/unique glycan profiles. The contribution of *N*-glycans to the biological function and structure of lactoferrin is not known.

Mammalian glycans are involved in multiple cellular mechanisms that are related to health and disease, and lactoferrin is no exception. Reports of a role for glycans in cell adhesion and receptor activation [60] strengthen the concept that the structure of glycoproteins is associated with the function of protecting the host against microbial and viral attacks. *N*-glycans also play roles in the recognition and association of microorganisms with cell membrane lectins [61]. The results of Barboza et al. [60], showing a dynamic variation of glycosylation as lactation progresses, support the hypothesis that human lactoferrin glycans are implicated in protecting the intestinal mucosa from pathogens encountered by the breastfed baby. Glycans also play a role in the behavior and structural properties of proteins [18]. A recent study reveals that *N*-glycans released from lactoferrin are involved in the inhibition of *P. aeruginosa*, bacteria responsible for bacterial keratitis and invasion of corneal epithelial cells [62]. Glycans determine much of protein folding and conformation, as well as greatly influencing protein solubility, immunogenicity, antigenicity, and the capacity to resist proteolysis [63]. The role of glycosylation in protein functions has not been elucidated. Improved understanding of the glycan structure of the polyfunctional protein lactoferrin offers another dimension for characterizing the relationship between the structure and function of proteins more broadly.

## 6. Deglycosylation Strategies to Study Protein Glycans

**Figure 5:**
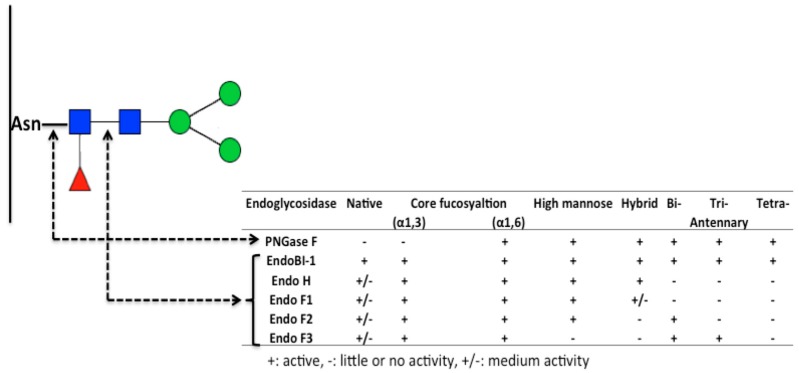
Endoglycosidases and their specificity on *N*-glycans.

The release of glycans from the polypeptide chain is a necessary step in the current generation of analytical methods used to characterize the glycosylated portions of proteins. Various chemical and enzymatically deglycosylation methods have been developed to release glycan moieties from glycoproteins. One of the commonly used chemical deglycosylation methods is hydrazine treatment. Patel et al. [64] used hydrazine treatment to selectively release *N*- and *O*-glycans from purified glycoprotein systems. Nevertheless, hydrazine poses health hazards, and is a carcinogen [65], and is therefore not suitable for food-related applications. To avoid these problems, *N*-Glycosidase F (PNGase F) is commonly used for enzymatic removal of *N*-glycans from milk glycoproteins, although this enzyme’s activity is very limited on native forms of glycoproteins [66]. Endoglycosidases F1, F2, and F3 are weakly active on native glycoproteins; however, the glycan structures which they can release are limited. Therefore, there is still a need for enzymes that are both active on native glycoprotein substrates, and can release a wide range of *N*-glycan structures. A recently described endo-β-*N*-acetylglucosaminidase (EndoBI-1) isolated from *Bifidobacterium longum* subsp. *infantis* ATCC 15697 cleaves the *N*,*N*′-diacetylchitobiose moiety at the *N*-glycan core, and its activity is not affected by core fucosylation. It also does not require substrate denaturation, enabling glycans in native form to be produced, so that their biological functions can be investigated using native state proteins. Various glysosidases and their activities on the *N*-glycan core in lactoferrin are shown in Figure 5.

## 7. Analytical Characterization of *N*-Glycans by Mass Spectrometry

Glycans are inherently the most potentially complex of the biomolecules, because their varied monosaccharide composition leads to multiple degrees of freedom in linkages and branching. The study of glycans requires the determination of their monosaccharide composition, and the information about their branching and isomeric (molecules with the same molecular formula but different chemical structures) and anomeric (isomers that only differ in the configuration of their anomeric carbon) configurations, which makes their analyses potentially more complex than other compounds, including DNA and proteins. During the past few decades, advances in glycobiology have allowed the development of many analytical techniques to solve this problem. One of the most sensitive and accurate methods to characterize oligosaccharides, including *N*-glycans, is modern mass spectrometry (MS).

Mass analysis by MS is now so accurate and sensitive that experiments can provide qualitative (structure) and quantitative (relative abundance) information on molecules after their conversion to ions [67,68]. Although several MS techniques exist, the underlying principle is the same. Methods for separation and analysis of *N*-glycans by high-pressure liquid chromatography coupled to MS (HPLC-MS) and tandem MS (MS/MS)—are discussed below.

### 7.1. N-Glycan Separation Using HLPC

HPLC separates molecules before their introduction to, and detection within the MS system. The combination of these two techniques allows the identification of molecules with identical composition, that differ only in the very precise geometry by which they are linked together. Specialized glycan-HPLC-chips are available to separate glycans at the isomer structural level. A glycan-HPLC-chip is composed of an enrichment column and a separation column packed with porous graphitized carbon that provides the stationary phase with affinity mechanisms that lead to the isomeric separation. The enrichment column accomplishes a refined miniaturized purification of a sample by trapping only the molecules of interest, and the separation column separates molecules based on their interaction with the stationary phase. A pump moves the sample solution with the liquid mobile phase in the graphitized carbon column that binds the *N*-glycans. Based on the strength of their partitioning between the mobile and the stationary phases, *N*-glycans are eluted at different times with different mobile phase (solvent) concentrations, allowing for their separation before detection. The nanospray tip produces a spray of protonated ions for MS analysis. New HPLC instruments, including nano-HPLC, have high sensitivity and reproducibility, and use only a small amount of sample (1–2 μL).

### 7.2. Mass Spectrometry

**Figure 6:**
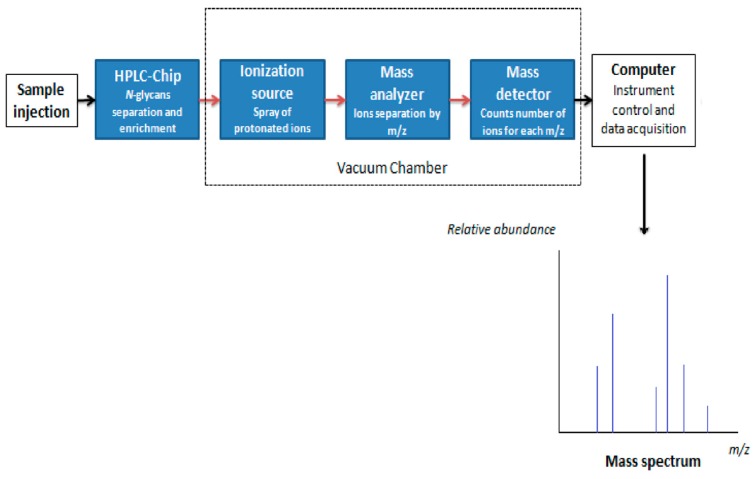
Components of a mass spectrometer.

Glycans are often ionized using a soft ionization method—electrospray ionization (ESI). The microfluidic chip with nano ESI is usually coupled to a high-accuracy quadrupole time-of-flight (QTOF) mass analyzer (Figure 6). With high precision, this measurement platform can elucidate complex mixtures molecule by molecule. The ions enter into the QTOF mass analyzer where they are accelerated by an electric field, and travel towards the detector as a function of their mass to-charge ratio. The QTOF mass spectrometer performs MS/MS using a quadrupole, a hexapole (collision cell), and a time-of-flight unit to produce spectra. The quadrupole selects precursor ions that are fragmented in the collision cell into product ions, which are then impelled to the detector at an angle perpendicular to the original path. All ions are given identical energy, and the time required to reach the detector is extremely sensitive to their mass-to-charge ratio (*m*/*z*). The detector counts the ions and translates the ionic energy in electrical energy. Signals are generated and recorded by a computer system that produces a mass spectrum showing the relative abundance of the detected compounds according to their *m*/*z* at very high speed and temporal accuracy. As a result, glycans can be rapidly analyzed in a high-throughput and reproducible manner.

### 7.3. Tandem Mass Spectrometry (MS/MS)

**Figure 7:**
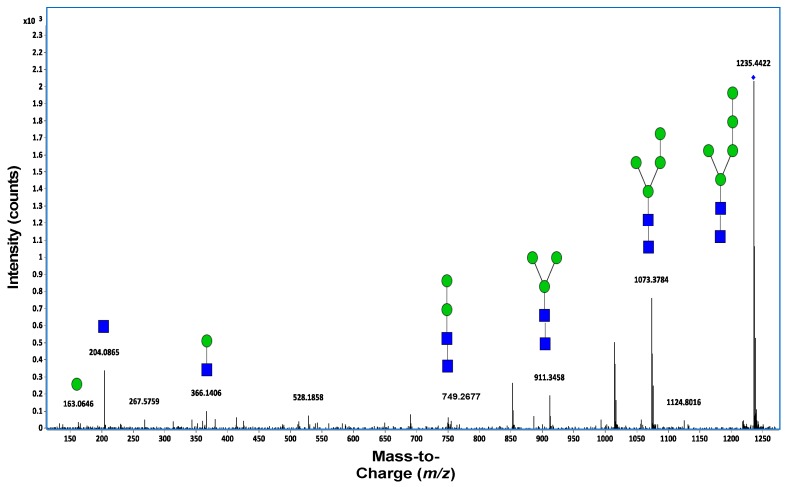
MS/MS spectra of a neutral lactoferrin *N*-glycan. Deconvoluted MS/MS spectrum of the neutral *N*-glycan 5Hex-2HexNAc. Green circles and blue squares represent mannose and HexNAc, respectively.

For particularly complex mixtures, measuring the accurate mass is not sufficient to identify structures. Therefore the MS/MS mode is used, where precursor ions are fragmented to generate product ions that are analyzed by QTOF. Fragmentation is performed by collision-induced dissociation (CID). Precursor ions strike collision gas molecules (nitrogen), leading to their fragmentation (Figure 7). These product ions give structural information about the initial molecule, enabling more accurate identification. *N*-glycans can initially be identified from the mass spectra based on accurate mass. MS/MS can be performed to confirm *N*-glycan compositions. MS/MS analysis generates specific fragment ions that are common to all *N*-glycans, including 163.06 *m*/*z* [Hex + H]^+1^, 204.09 *m*/*z* [HexNAc + H]^+1^, and 366.14 *m*/*z* [HexNAc − Hex + H]^+1^. Each spectrum can be screened for the presence of fragment ions. After *N*-glycan identification, molecules can be entered into digital data libraries. These libraries typically include mass, retention time, and monosaccharide compositions. The availability of these libraries has driven the development of software programs capable of interrogating these libraries in real time to identify molecules in chromatograms based on the accurate mass and retention time. Hua and Kronewitter [69,70] established, for mice and humans, respectively, libraries of *N*-linked glycans, illustrating the enabling utility of this approach.

## 8. Conclusions

Research in multiple disciplines interfacing glycobiology continues to discover novel biological roles for the multi-functional protein lactoferrin. It is, therefore expected that the diverse structures and functions of lactoferrin will remain a popular target for investigations, and that this research will reveal additional functions and health benefits in the near future. While the backbone sequence of milk proteins has been extensively elucidated via proteomics studies, the roles of glycosylation in contributing to, or altering these protein bioactivities, have largely been ignored. Advancements in cataloguing the complexity and unique glycosylation patterns of lactoferrin in different biological sources are necessary to for next phase of research: illuminating the exceptional contribution of glycans to biological and physicochemical properties of a wide range of proteins. The application of novel deglycosylating enzymes will play important roles for the determination of the contribution of these glycans to the function of lactoferrin. Especially, considering EndoBI-1’s ability to release specific glycan structures at different conditions, this will enable the discrimination of the activity of multiple glycans pools. Moreover, the application of novel enzymes will help to determine the actual sites of *N*-glycans for which very limited information is currently available. The combination of these novel approaches with advanced mass spectrophotometric tools and bioinfomatic libraries, will enable structure-activity studies to be made of the naked protein backbone and the glycosylated form, and these technologies will enable the identification of key specific glycan compositions for functional activities. Numerous products are already on the market, or under development.

## Abbreviations

Asn	Asparagine
bLF	Bovine lactoferrin
CID	Collision-induced dissociation
ESI	Electrospray ionization
Fuc	Fucose
GlcNAc	*N*-acetylglucosamine
Hex	Hexose
hLF	Human lactoferrin
HPLC-MS	High-pressure liquid chromatography coupled to mass spectrometry
MS	Mass spectrometry
MS	Mass spectrometry
NeuAC	Sialic acid
NeuGc	*N*-glycolylneuraminic acid
QTOF	Quadrupole time-of-flight
rhLF	Recombinant human lactoferrin
